# Supplementary figures and images for: Oligoclonal IgG antibodies in multiple sclerosis target patient-specific peptides
Source: PLoS One. 2020 Feb 21;15(2):e0228883. doi: 10.1371/journal.pone.0228883 (PMC7034880; doi:10.1371/journal.pone.0228883)

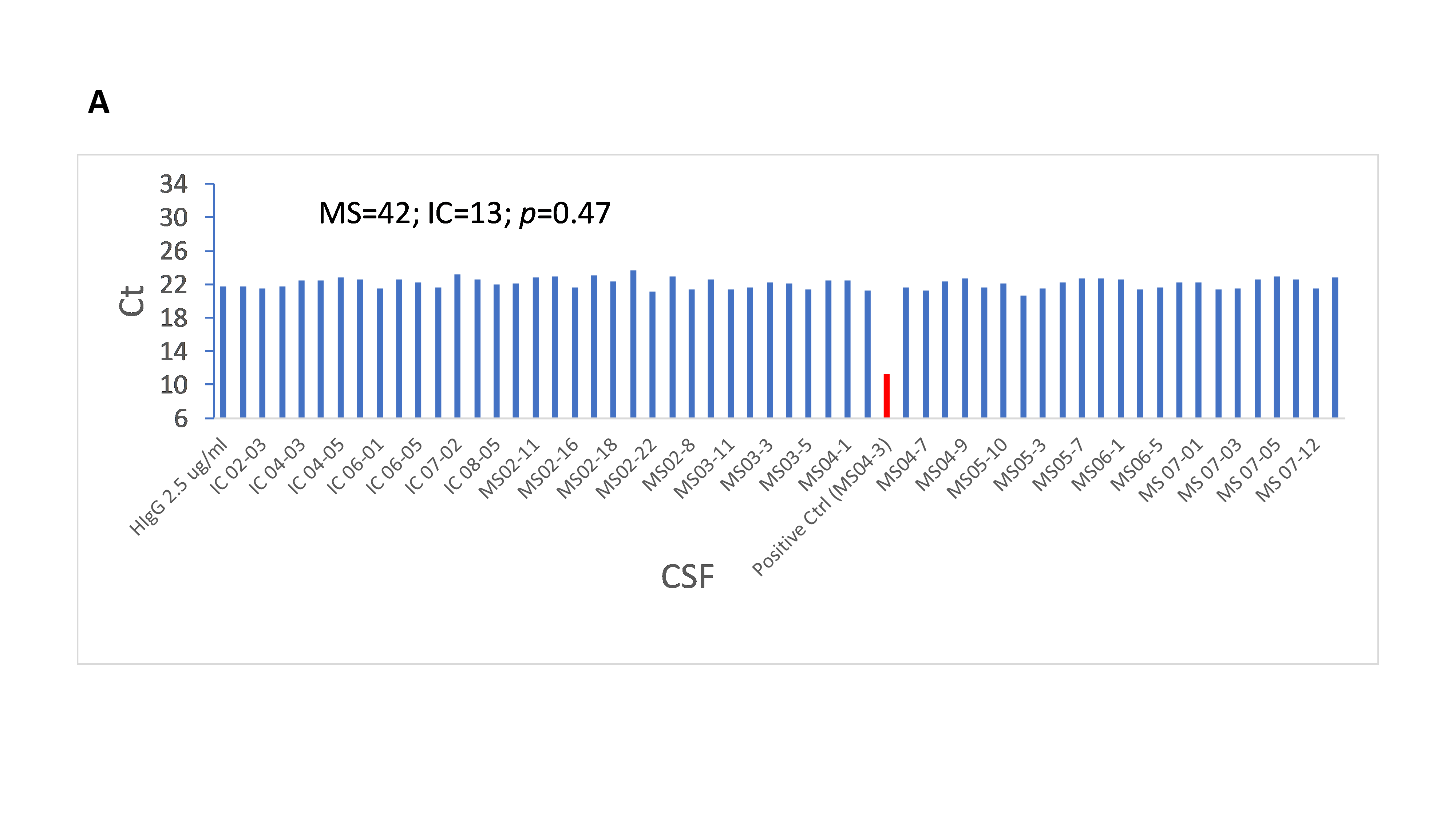

Supplement: S1 Fig — A. There is no shared and differential phage peptide binding between MS and IC CSF. IPCR was performed to screen 42 MS and 13 IC CSF with pooled phage peptides MS05-4A6, MS05-4A2, and MS04-3B1. p = 0.47. B. Summary of S1 Fig. A. There is no shared and differential phage peptide binding between MS and IC CSF. (TIF) [file pone.0228883.s001.tif]
