# Supplementary material for: Oligoclonal IgG antibodies in multiple sclerosis target patient-specific peptides
Source: PLoS One. 2020 Feb 21;15(2):e0228883. doi: 10.1371/journal.pone.0228883 (PMC7034880; doi:10.1371/journal.pone.0228883)
Supplement: S1 Table — (DOCX) [file pone.0228883.s002.docx]

**Table 1. MS CSF used for IPCR screening.**

| **Sample #** | **CSF IgG (µg/mL)** | **Diagnosis** | **Sex** | **% IgG^1^** | **OCBs^2^** |
| --- | --- | --- | --- | --- | --- |
| MS02-10 | 67 | RRMS | F | 19.00 | 2 |
| MS02-11 | 71 | RRMS | F | 21.00 | 3 |
| MS02-13 | 41 | RRMS | F | 13.00 | 2 |
| MS02-16 | 14 | RRMS | F | 6.00 | 0 |
| MS02-17 | 30 | RRMS | F | 13.00 | 6 |
| MS02-18 | 33 | RRMS | F | 17.00 | 3 |
| MS02-2 | 28 | RRMS | M | 7.00 | 0 |
| MS02-22 | 107 | RRMS | F | 27.00 | 4 |
| MS02-7 | 41 | RRMS | F | 10.00 | 4 |
| MS02-8 | 26 | RRMS | F | 11.00 | 3 |
| MS03-10 | 39 | RRMS | F | 17.00 | 5 |
| MS03-11 | 53 | RRMS | F | 18.00 | 7 |
| MS03-13 | 107 | RRMS | F | 17.00 | 5 |
| MS03-3 | 24 | RRMS | M | 7.00 | 0 |
| MS03-4 | 64 | RRMS | F | 27.00 | 4 |
| MS03-5 | 51 | RRMS | F | 15.00 | 6 |
| MS03-7 | 86 | RRMS | F | 32.00 | 3 |
| MS04-1 | 37 | RRMS | F | 9.00 | 4 |
| MS04-2 | 219 | PPMS | F | 21.00 | 5 |
| MS04-4 | 18 | RRMS | F | 8.50 | + |
| MS04-7 | 31 | PPMS | F | 14.10 | + |
| MS04-8 | 39 | RRMS | F | 12.10 | + |
| MS04-9 | 28 | RRMS | M | 7.20 | 0 |
| MS05-1 | 92 | RRMS | M | 23.00 | + |
| MS05-10 | 88 | RRMS | F | 20.90 | 28 |
| MS05-2 | 72 | RRMS | F | 24.80 | 19 |
| MS05-3 | 57 | RRMS | F | 23.70 | 21 |
| MS05-5 | 38 | RRMS | M | 9.00 | 16 |
| MS05-7 | 98 | RRMS | M | 18.40 | 8 |
| MS05-8 | 112 | RRMS | F | 22.40 | 19 |
| MS06-1 | 25 | RRMS | M | 7.40 | 12 |
| MS06-4 | 20 | RRMS | F | 9.40 | 14 |
| MS06-5 | 35 | RRMS | M | 8.80 | 3 |
| MS 06-06 | 127 | RRMS | F | 33.4 | 19 |
| MS 07-01 | 37 | RRMS | F | 16.8 | 14 |
| MS 07-02 | 48 | RRMS | F | 13 | 22 |
| MS 07-03 | 19 | RRMS | F | 6.8 | 8 |
| MS 07-04 | 90 | RRMS | M | 14.8 | 19 |
| MS 07-05 | 14 | RRMS | F | 6.4 | 1 |
| MS 07-09 | 44 | RRMS | F | 7.6 | ND |
| MS 07-12 | 33 | RRMS | F | 11 | 13 |
| MS 07-14 | 72 | RRMS | F | 8.8 | 12 |

1. % IgG: Percent of IgG of total protein in CSF.
2. Number of OCBs present.
