# Supplementary material for: Oligoclonal IgG antibodies in multiple sclerosis target patient-specific peptides
Source: PLoS One. 2020 Feb 21;15(2):e0228883. doi: 10.1371/journal.pone.0228883 (PMC7034880; doi:10.1371/journal.pone.0228883)
Supplement: S2 Table — (DOCX) [file pone.0228883.s003.docx]

| **Sample #** | **CSF IgG (µg/mL)** | **Diagnosis** | **Sex** | **% IgG^1^** | **OCBs** |
| --- | --- | --- | --- | --- | --- |
| IC 02-03 | ND^2^ | Acute viral meningitis | F | ND | ND |
| IC 04-01 | 12 | Behcet's disease | F | 5.4 | 0 |
| IC 04-03 | 9 | Paraneoplastic syndrome | F | ND | 0 |
| IC 04-04 | 678 | Viral meningitis | M | 36.8 | + |
| IC 04-05 | 552 | Cryptococcal meningitis | M | ND | + |
| IC 05-02 | 85 | Chronic meningitis of unknown etiology | M | 20.2 | 21 |
| IC 06-01 | 386 | Subacute sclerosing panencephalitis | M | 59.2 | 17 |
| IC 06-04 | 38 | ADEM Acute disseminated encephalomyelitis) | F | 9.3 | 2 |
| IC 06-05 | 180 | Paraneoplastic encephalitis | M | 24 | 23 |
| IC 07-01 | 45 | Neurosyphilis | M | 8.5 | 0 |
| IC 07-02 | 79 | Chronic progressive meningoencephalitis | M | 11.7 | 7 |
| IC 08-04 | 54 | VZV myelopathy | M | ND | 1 |
| IC 08-05 | 26 | VZV radiculomyelitis | F | ND | 0 |

**Table 2. Inflammatory control (IC) patients used for IPCR CSF screening.**

1. % IgG: Percent of IgG of total protein in CSF.
2. ND: not determined.
