# Supplementary material for: Oligoclonal IgG antibodies in multiple sclerosis target patient-specific peptides
Source: PLoS One. 2020 Feb 21;15(2):e0228883. doi: 10.1371/journal.pone.0228883 (PMC7034880; doi:10.1371/journal.pone.0228883)

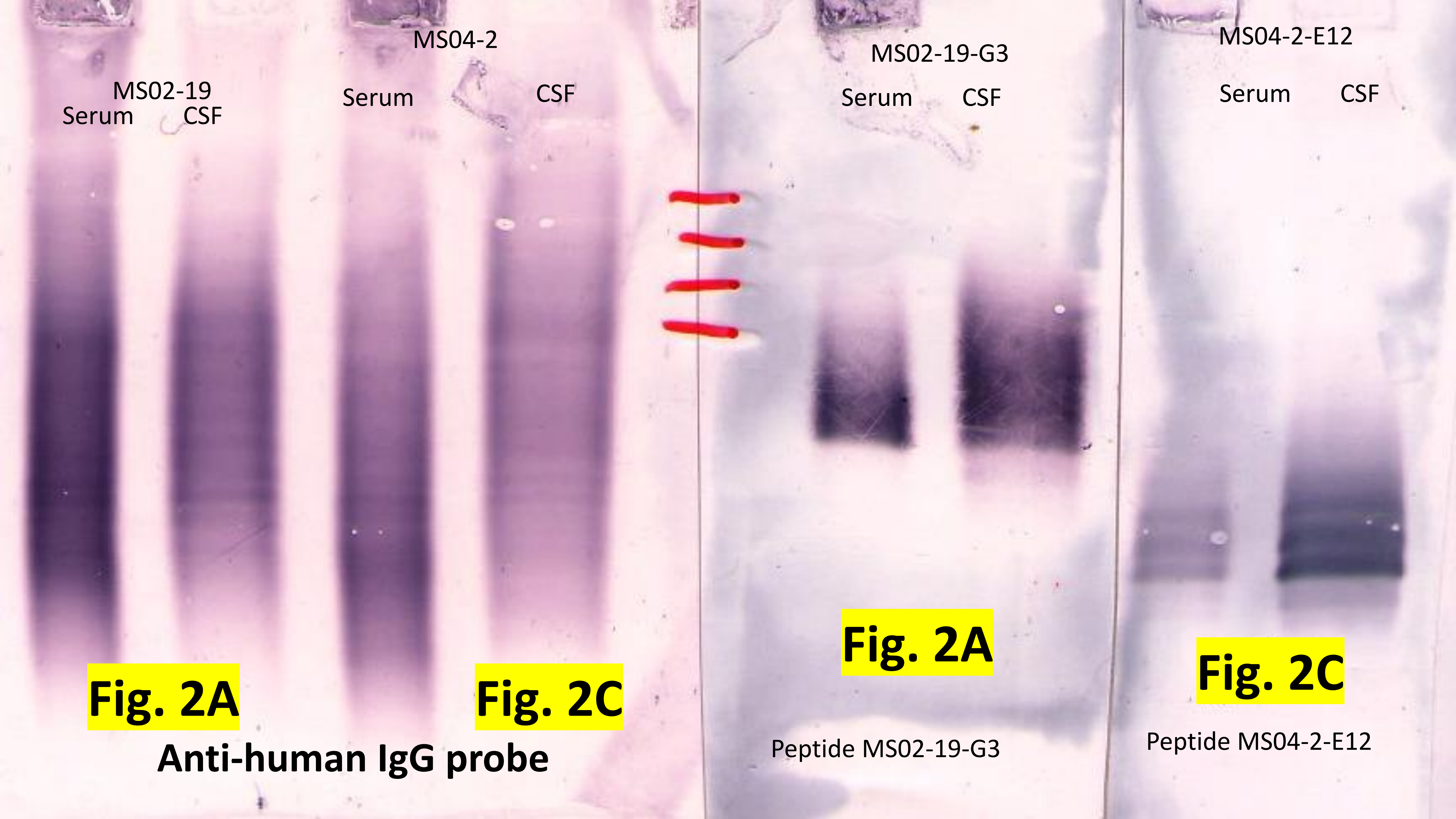
MS02-19
Serum
CSF
MS04-2
Serum
CSF
MS02-19-G3
Serum
CSF
MS04-2-E12
Serum
CSF
Fig. 2A
Fig. 2C
Anti-human IgG probe
Fig. 2A
Peptide MS02-19-G3
Fig. 2C
Peptide MS04-2-E12

Peptide MS03-7A2

Fig. 2B

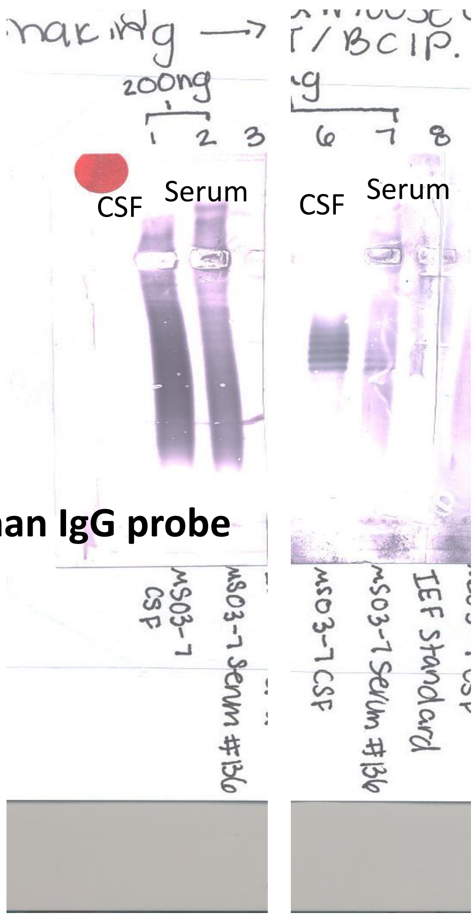

Anti-human IgG probe

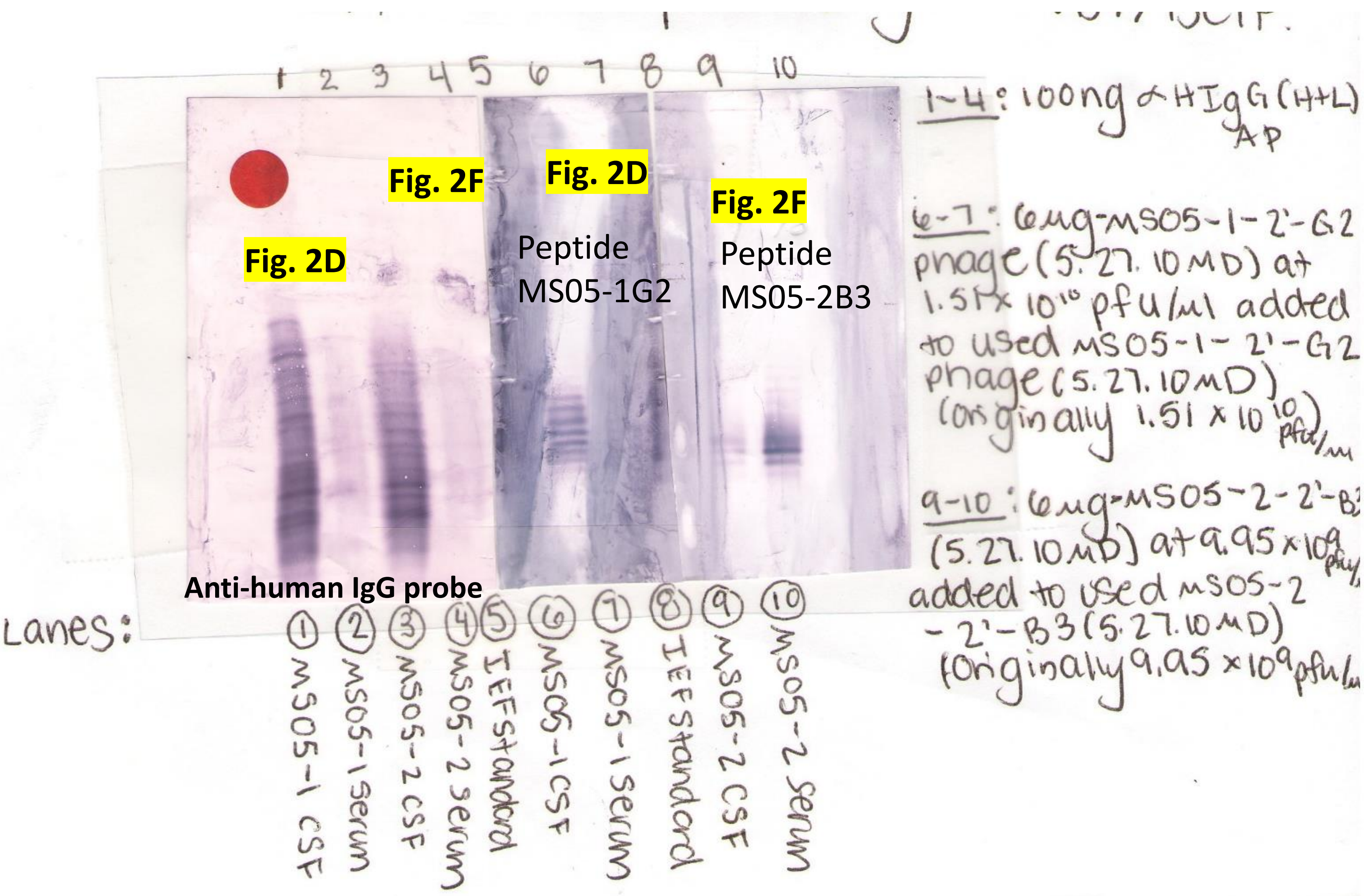

1 2 3 4 5 6 7 8 9 10
Fig. 2F
Fig. 2D
Fig. 2F
Fig. 2D
Peptide MS05-1G2
Peptide MS05-2B3
Anti-human IgG probe
1-4: 100ng αHIgG(H+L) AP
6-7: 6ug-MS05-1-2'-G2 phage (5.27.10MD) at 1.51 x 10^10 pfu/ml added to used MS05-1-2'-G2 phage (5.27.10MD) (originally 1.51 x 10^10 pfu/ml)
9-10: 6ug-MS05-2-2'-B3 (5.27.10MD) at 9.95 x 10^9 pfu/ml added to used MS05-2-2'-B3 (5.27.10MD) (originally 9.95 x 10^9 pfu/ml)
Lanes:
1 MS05-1 CSF
2 MS05-1 serum
3 MS05-2 CSF
4 MS05-2 serum
5 IEF standard
6 MS05-1 CSF
7 MS05-1 serum
8 IEF standard
9 MS05-2 CSF
10 MS05-2 serum

Fig. 2E

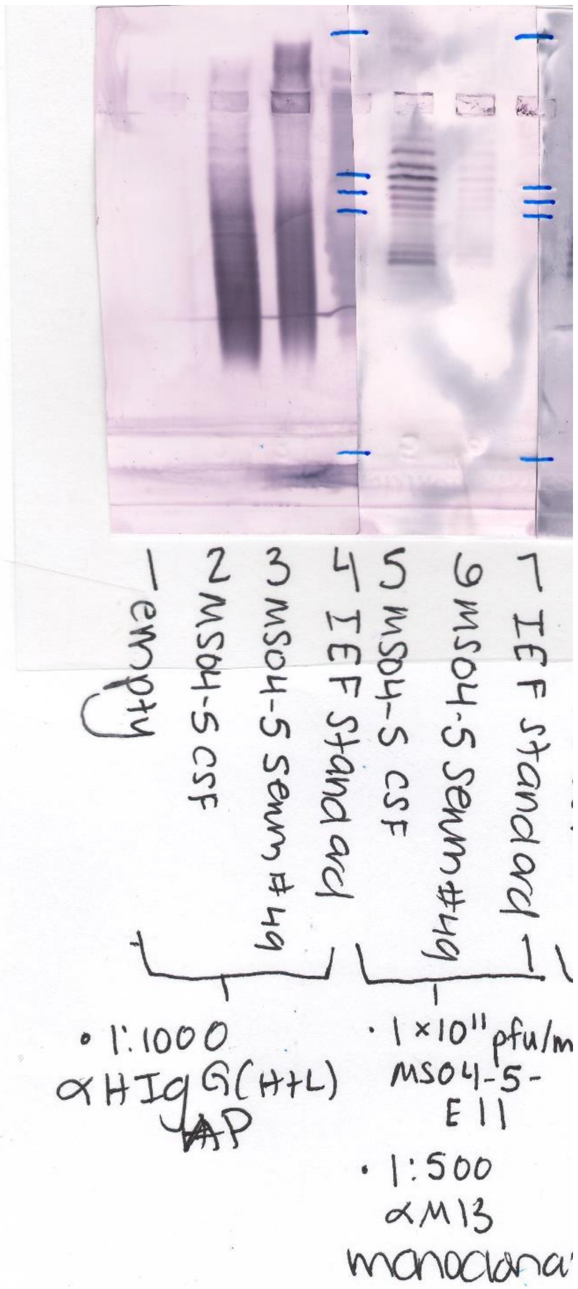

1 empty
2 MS04-5 CSF
3 MS04-5 serum #49
4 IEF standard
5 MS04-5 CSF
6 MS04-5 serum #49
7 IEF standard
• 1:1000
αHIgG(H+L)
AP
• 1×10¹¹ pfu/m
MS04-5-
E11
• 1:500
αM13

Fig. 2G

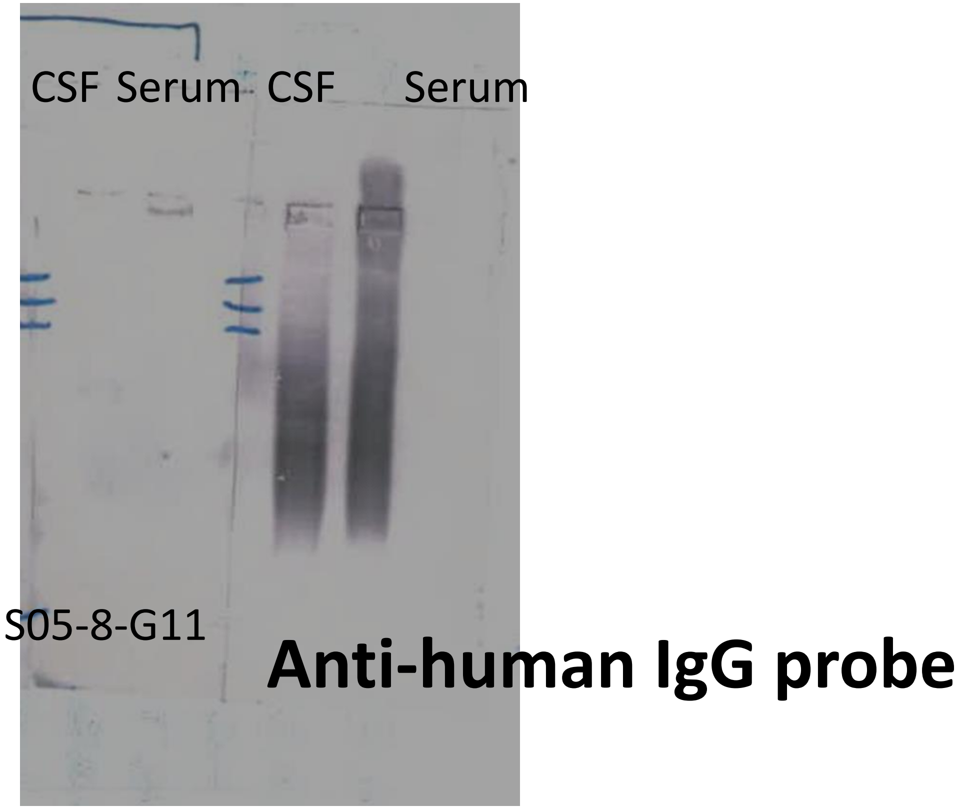

Fig. 3A MS #7 phage (MS04-3B1)

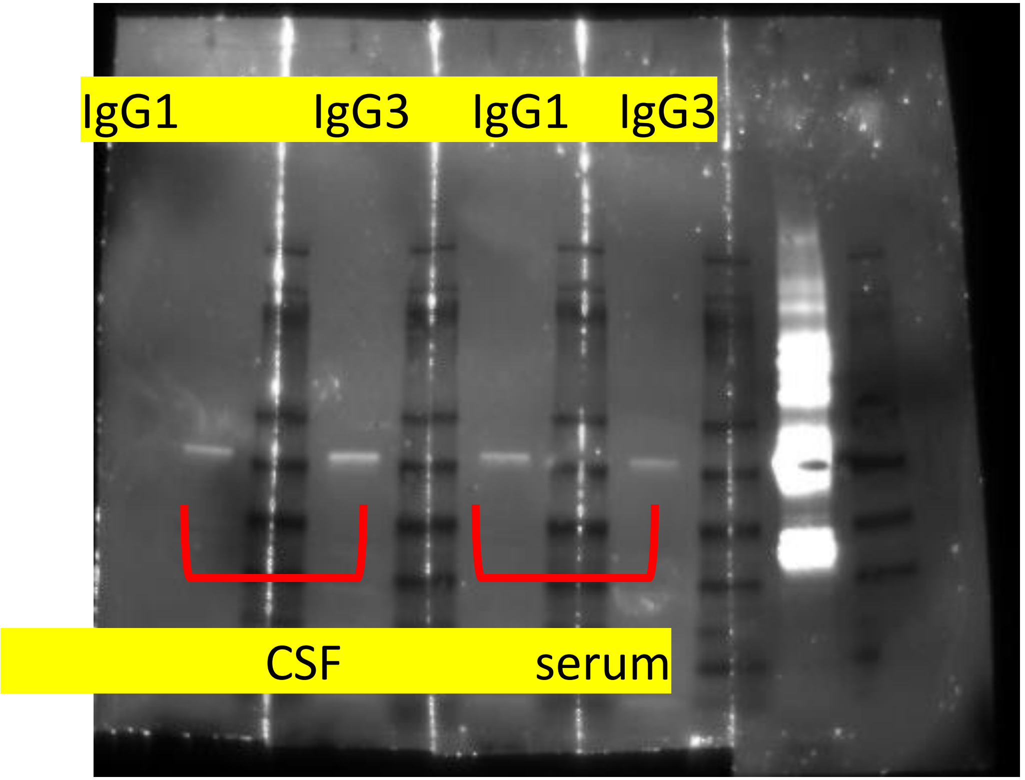

Fig. 3A MS #13 phage (MS07-12-B11*)

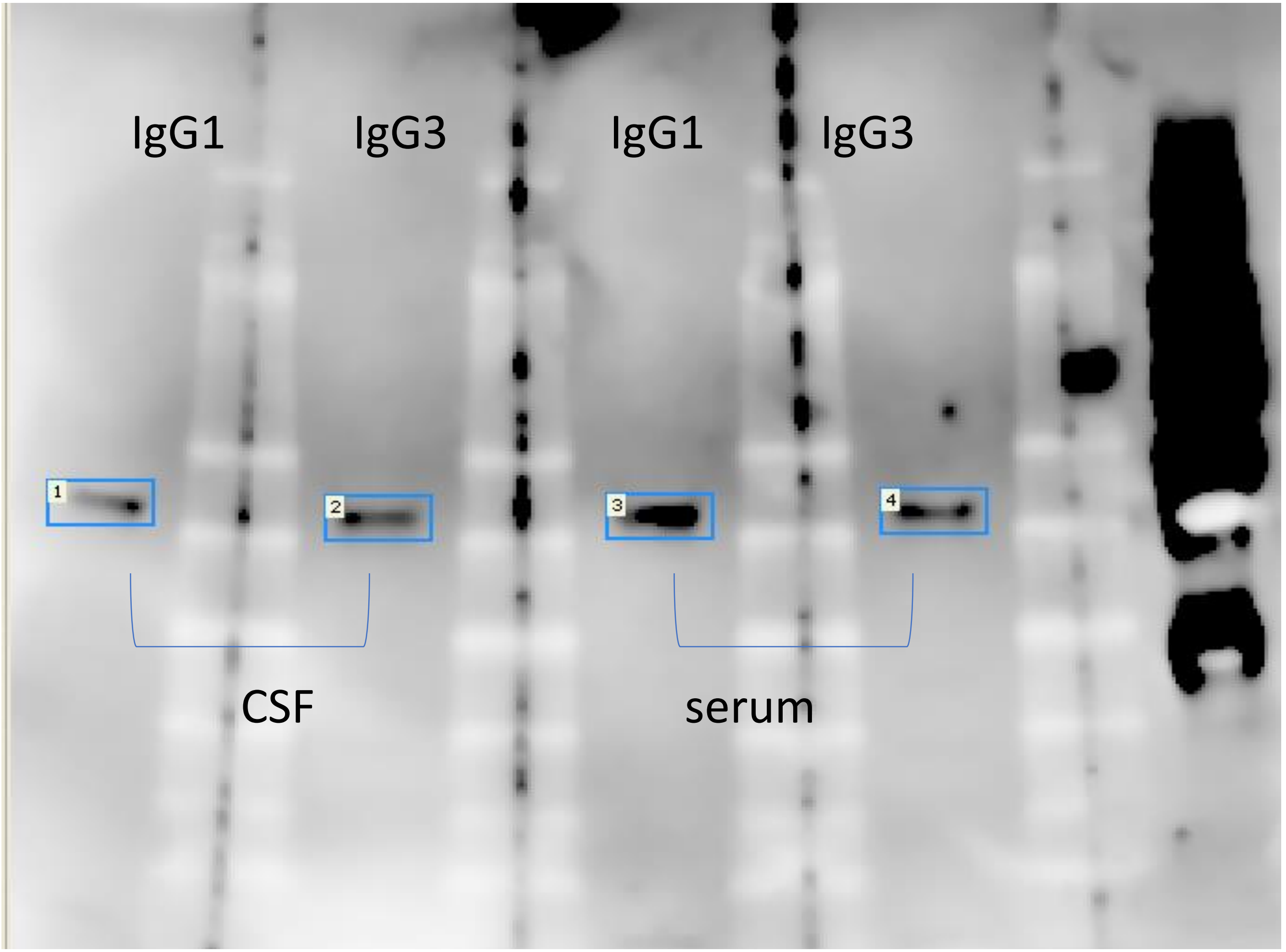

Supplement: S1 Raw Images — (PDF) [file pone.0228883.s004.pdf]
